# S100A expression in normal corneal-limbal epithelial cells and ocular surface squamous cell carcinoma tissue

**Published:** 2011-08-20

**Authors:** Jing Li, Andri K. Riau, Melina Setiawan, Jodhbir S. Mehta, Seng-Ei Ti, Louis Tong, Donald T.H. Tan, Roger W. Beuerman

**Affiliations:** 1 Department of Ophthalmology, Xinhua Hospital, Shanghai Jiao Tong University, Shanghai, China; 2 Singapore Eye Research Institute, Singapore; 3 Department of Ophthalmology, Yong Loo Lin School of Medicine, National University of Singapore, Singapore; 4 Singapore National Eye Centre, Singapore; 5 Department of Clinical Sciences, Duke-NUS Graduate Medical School, Singapore; 6 SRP Neuroscience and Behavioral Disorders, Duke-NUS Graduate Medical School, Singapore

## Abstract

**Purpose:**

To study the expression and cellular distribution of multiple S100A genes and proteins in normal corneal-limbal epithelium and ocular surface squamous cell carcinoma (SCC) tissue.

**Methods:**

Normal corneal-limbal tissue was obtained from the Lions Eye Bank, Tampa, FL. Ocular surface SCC tissues were excised from patients undergoing surgery at Singapore National Eye Centre. *S100A* mRNA expression was measured by quantitative PCR. S100 protein distribution was determined by immunofluorescent staining analysis.

**Results:**

Twelve *S100* mRNAs were identified in human corneal and limbal epithelial cells. *S100A2*, A6, A8, A9, A10, and A11 mRNA was expressed at high level, while *S100A1*, A3, A4, A5, A6, A7, and A12 mRNA expression was low. The intracellular localization of S100A2, A6, A8, A9, A10 and A11 protein was determined in normal corneal-limbal and SCC tissues. S100A2 and S100A10 proteins were enriched in basal limbal epithelial cells of the normal tissue. S100A8 and S100A9 were found only at the surface of peripheral corneal and limbal epithelium. S100A6 was uniformly found at the plasma membrane of corneal and limbal epithelial cells. S100A11 was found at the supralayer limbal epithelial cells adjacent to the conjunctiva. SCC tissue showed typical pathological changes with expression of cytokeartin (CK) 14 and CK4 in the epithelial cells. All SCC epithelial cells were positive of S100A2, S100A10, S100A6 and S100A11 staining. Intracellular staining of S100A8 and S100A9 was found in several layers of SCC epithelium. Expression of S100A2 and S100A10 decreased dramatically in cultured limbal epithelial cells with increased passaging, which was accompanied by a small increase of *S100A9* mRNA, with no changes of *S100A8* gene expression. Serum and growth hormone depletion of the culture serum caused a small reduction of *S100A2* and *S100A10* gene expression, which was accompanied by a small increase of *S100A9* mRNA while no changes of *S100A8* expression was measured.

**Conclusions:**

Normal corneal and limbal epithelial cells express a broad spectrum of *S100* genes and proteins. Ocular surface SCC express high levels of S100A2, S100A10, S100A8 and S100A9 proteins. The expression of S100A2 and S100A10 is associated with limbal epithelial cell proliferation and differentiation.

## Introduction

S100 proteins are a group of small acidic proteins of 10–12 kDa [1]. With more than 20 proteins identified, they form the largest family of calcium binding proteins. Each S100 protein has two calcium-binding EF-hand motifs: a modified S100-specific EF hand at the NH_2_-terminus and a classical one at the COOH-terminus. The two EF-hand motifs are connected by a central hinge sequence. Upon calcium binding, the hinge region undergoes large reorientation and exposes the binding interface for its target proteins such as annexins, cytoskeleton proteins, p53, and pattern recognition receptors [2-5]. Through binding with different proteins, S100 proteins are involved in the regulation of many important cellular activities such as calcium homeostasis, cytoskeleton organization, stress response, cell motility, cell proliferation and differentiation. Most noticeably, abnormal expression of many S100 proteins, such as S100A2, S100A4, S100A6, S100A8, S100A9, S100A10, and S100A11 is found in numerous cancers [6].

Several studies have reported the expression of S100 proteins in the ocular tissue. For example, abnormal S100A2 and S100A4 expression was found in human keratoconus tissue [7,8]. S100A4 and S100B proteins were found in activated stromal myofibroblast after corneal debridement, likely involved in stromal cell proliferation and wound healing [9,10]. A recent study reported the role of neutrophil secreted S100A8 and A9 proteins in mouse models of corneal neovascularization [11]. Upregulation of multiple other *S100* mRNA expression such as *S100A4*, *S100A6*, and *S100A13* was reported in the study [11]. However, the cellular source for these gene products was unclear. We have previously reported increased expression of *S100A6*, *S100A8*, and *S100A9* mRNA and protein in pterygial tissue compared to normal conjunctiva [12]. Increased concentration of S100A8 and S100A9 was also detected in pterygium patient tear samples compared to healthy controls [13]. In another study, we reported increased S100A4, S100A8, S100A9, and S100A11 proteins in tear samples obtained from dry eye patients [14]. Collectively, these studies suggest the involvement of multiple S100A proteins in inflammatory and proliferative conditions of the ocular surface. However, the scope of *S100A* gene and protein expression in human corneal cells remains unknown.

Ocular surface squamous cell carcinoma (SCC) is one of the major cause for ocular morbidity and mortality. It is featured by dysregulated proliferation and differentiation of corneal and conjunctival epithelial cells [15]. A benign form of proliferative disorder of the ocular surface is the corneal/conjunctival intraepithelial neoplasm (CIN) [16]. Despite the extensive report on S100 proteins in various cancers, the involvement of S100 proteins in these conditions is unknown. Here we report the differential expression and cellular distribution of multiple *S100A* genes and proteins in normal corneal-limbal and ocular surface SCC epithelial cells. We further demonstrate the association between limbal epithelial cell differentiation and the expression of *S100A2* and *S100A10* genes. Our results suggest that selective S100 proteins are involved in corneal epithelial cell proliferation and differentiation under both normal and pathological conditions.

## Methods

### Human corneal and limbal epithelial cell isolation and culture

Cadaver corneal-limbal tissues were obtained from the Lions Eye Bank, Tampa, Florida. The corneal epithelial cells were collected by scraping the corneal surface using a sterile surgical blade. The blade was rinsed with 1 ml of Trizol solution immediately and RNA was extracted. The peripheral/limbal region 2–3 mm inside the thin circle of pigmentation was avoided during the scraping. After the scraping, the remaining limbal rim was excised, washed with antibiotics and subjected to dispase followed by trypsin digestion. Detailed protocols for the isolation and culture of human limbal epithelial cells have been published previously by our group [17,18]. Isolated limbal epithelial cells were cultured in SHEM medium which contained equal volumes of DMEM and F12, 2 ng/ml of recombinant human epidermal growth factor (EGF), 1 μg/ml bovine insulin, 0.1 μg/ml cholera toxin, 0.5 μg/ml hydrocortisone, and 10% fetal bovine serum (FBS) in the presence of mitomycin-C inactivated 3T3 fibroblasts. Limbal cells were passaged when more than 70% of the culture dish area was covered by colonies and the majority of the colonies had about 100–200 cells. For serum and growth factor depletion experiment, the limbal epithelial cells were cultured in the above medium without EGF, insulin, and FBS for 24 h.

### Reverse transcription (RT) and quantitative PCR (qPCR) analysis

PureLink (Invitrogen, Singapore) was used for total RNA extraction. RNA concentration was evaluated by Nanodrop spectrophotometer. RNA (250 ng) was reverse transcribed to cDNA using RTIII (Invitrogen, Singapore). cDNA (0.5 μl) was used in SYBR-green based qPCR analysis using Roche LightCycler 480 and Roche PCR master mix (Roche, Singapore). Primer sequences for individual *S100A* mRNAs are listed in Table 1. A single peak of qPCR product with the melting temperature between 80 and 90 °C was observed for each reaction. Glyceraldehyde 3-phosphate dehydrogenase (*GAPDH*) and β-actin (*ACTB*) were used as internal controls for the calculation of relative cycle threshold (ΔCp; ΔCp=Cp [target] – Cp [*ACTB*] or Cp [*GAPDH*]). Each cDNA sample was analyzed in triplicates. Fold changes was calculated as 2^- ΔΔCp^ relative to *S100A4* in corneal cells.

**Table 1:** Primers used for *S100A* qPCR.

**Gene name**	**Primer sequences (5′-3′)**	**Amplicon size (bp)**	**Annealing temperature**
*S100A1*	F: CTTGGCCATCTGTCCAGAAC		
* *	R: CAATGTGGCTGTCTGCTCAA	362	56 °C
*S100A2*	F: GCCAAGAGGGCGACAAGTT		
* *	R: AGGAAAACAGCATACTCCTGGA	175	60 °C
*S100A3*	F: GCGGTAGCTGCCATCGTGTG		
* *	R: GCAGTCCTTGAAGTACTCGT	258	56 °C
*S100A4*	F: GATGAGCAACTTGGACAGCAA		
* *	R: CTGGGCTGCTTATCTGGGAAG	123	60 °C
*S100A5*	F: CTGCACACTGTGATGGAGAC		
* *	R: GAAGTCGTTGTAGGCCATGC	270	56 °C
*S100A6*	F: AAGCTGCAGGATGCTGAAAT		
* *	R: CCCTTGAGGGCTTCATTGTA	131	56 °C
*S100A7*	F: TGCTGACGATGATGAAGGAG		
* *	R: ATGTCTCCCAGCAAGGACAG	131	56 °C
*S100A8*	F: ATGCCGTCTACAGGGATGAC		
* *	R: ACGCCCATCTTTATCACCAG	160	56 °C
*S100A9*	F: GTGCGAAAAGATCTGCAAAA		
* *	R: TCAGCTGCTTGTCTGCATTT	103	56 °C
*S100A10*	F: ATGCCATCTCAAATGGAACA		
* *	R: CTACTTCTTTCCCTTCTGCT	294	56 °C
*S100A11*	F: ATGGCAAAAATCTCCAGCCC		
* *	R: TCATCATGCGGTCAAGGACA	193	56 °C
*S100A12*	F: CCTCTCTAAGGGTGAGCTGA		
* *	R: CTGGGTTTTGGTGAGGGAAA	271	56 °C
*ACTB*	F: ATCATGTTTGAGACCTTCAACA		
* *	R: CATCTCTTGCTCGAAGTCCA	318	56 °C
*GAPDH*	F: CCATGTTCGTCATGGGTGTGAACGA		
* *	R: GCCAGTAGAGGCAGGGATGATGTTC	254	56 °C

### SCC patient information

Ocular surface SCC tissues were obtained from 2 patients who underwent surgical excision. The first case was a 60-year-old Chinese male with recurrent corneal/conjunctival intraepithelial neoplasia for 5 years that developed into SCC. The second case was another 60-year-old Chinese male with a left eye SCC who originally presented with sclerokeratitis for 8 months with corneal scarring. The appearance of a large nodule nasal to the area of sclera thinning appeared on the 8th month and a diagnosis of SCC was made following excision biopsy. No signs of tumor recurrence were noticed 5 months after the excision surgery.

Informed written consent was obtained from each participant. This study was performed in accordance with the tenets of the Declaration of Helsinki and the study protocol was approved by SingHealth Institutional Review Board.

### Histological and immunofluorescent staining analysis

Histological analysis of SCC biopsy and immunofluorescent staining of S100 proteins of SCC and normal corneal and limbal tissues were performed as previously described [12]. The slides were fixed with freshly made 4% paraformaldehyde (PFA), blocked by 4% BSA with 0.1% Triton X-100 and incubated with the following antibodies overnight at 4 °C: mouse monoclonal anti-S100A2 (clone SH-L1; Sigma, Singapore) antibody at 1:1000 dilution; mouse monoclonal anti-S100A6 antibody (6B5; Abnova, Taipei, Taiwan) at 1:300 dilution; mouse monoclonal anti-S100A8 antibody (8–5C2; Acris, Herford, Germany) at 1:150 dilution; mouse monoclonal anti-S100A9 antibody (1C10; Abnova) at 1:150 dilution; rabbit polyclonal anti-S100A10 (Abcam, Cambridge, MA) at 1:100 dilution; mouse monoclonal anti-S100A11 antibody (2F4; Abnova) at 1:150 dilution; mouse monoclonal anti-CK3/12 antibody (2Q1040 Abcam) at 1:50 dilution; mouse monoclonal anti-CK4 antibody (6B10 Acris) at 1:50 dilution; and goat polyclonal anti-CK14 antibody (Santa Cruz Biotechnology, Santa Cruz, CA) at 1:50 dilution. This was followed by incubation with an Alexa Fluor 488-conjugated secondary antibody for 1 h and the slides were mounted with DAPI-containing UltraCruz Mounting Medium (Santa Cruz Biotechnology). The slides were examined under a Zeiss Axioplan 2 fluorescence microscope (Zeiss, Gottingen, Germany) and digital images of representative areas were taken.

### Western blot analysis of cytokeratin 3 protein

Cultured human primary limbal epithelial cells were washed with 2 mM EDTA followed by PBS to remove 3T3 feeder cells and lysed with RIPA buffer [18]. Total protein (20 µg) was loaded on SDS–PAGE and transferred to nitrocellulose paper. The blot was probed by an anti-cytokeratin 3 antibody (AE5; Santa Cruz Biotechnology, Santa Cruz, CA) and a major band of 64 kDa (cytokeratin 3) was detected.

## Results

### Expression of S100 genes in human corneal and limbal epithelial cells

**Figure 1:**
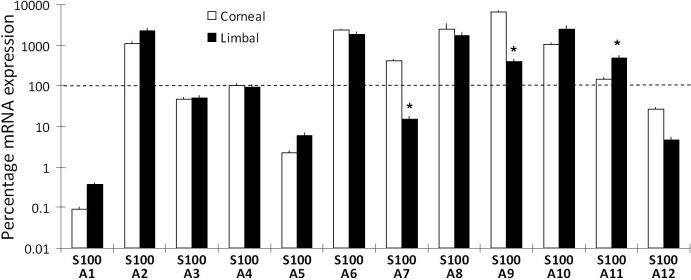
Relative abundance of *12 S100A* mRNA in human corneal and primary cultured limbal epithelial cells. The data represents averaged value from 6 different preparations of limbal and 3 corneal epithelial cells. For each cDNA samples, qPCR was performed in triplicates. ΔCp was calculated against β-actin (*ACTB*). The ΔCp of *S100A4* in corneal cells was taken as 100% and used as reference for the calculation of the relative abundance of other *S100A* mRNA. Error bars stand for the standard deviation of the averaged results. *Represents statistically significant difference (p<0.05 by unpaired *t*-test) between corneal and limbal epithelial cells for the same mRNA.

Expression of *S100A1*, A2, A3, A4, A5, A6, A7, A8, A9, A10, A11 and A12 mRNA was measured by qPCR. The levels of individual mRNA expression in corneal (3 samples) and limbal epithelial cells (6 samples) were calibrated using *ACTB* as an internal control and expressed relative to *S100A4* in corneal cells (Figure 1). Similar results were obtained using *GAPDH* as an internal control (Data not shown). Between corneal and limbal epithelial cells, *S100A7* and *S100A9* mRNA was more abundant in corneal epithelial cells while *S100A11* mRNA was more abundant in cultured limbal epithelial cells (p<0.05, unpaired *t*-test).

### Histological analysis and cytokeratin (CK) expression in ocular surface SCC epithelial cells

**Figure 2:**
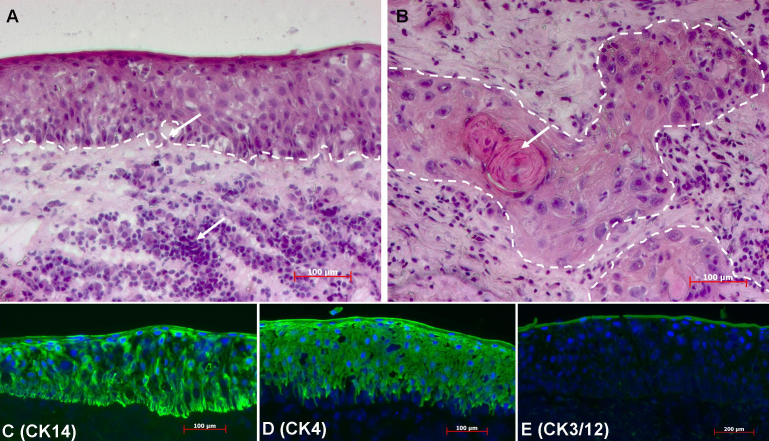
Morphological features and cytokeratin protein expression in SCC tissue. **A** and **B**: H&E staining of SCC tissue. **A**: Notice the thickening of the epithelium as demarcated by the white dotted line, the breach of the basement membrane and massive leukocytes infiltration (white arrows). **B**: The epithelial cell invasion of the stroma (marked by the dotted white lines). The keratin pearls formed by necrotic epithelial cells (white arrow). The invading cells showed typical metaplastic appearance with enlarged nucleus and cell volume. The original pictures were taken at 100× magnification. **C** and **D**: Immunofluorescent staining of cytokeratin proteins (green) in SCC tissue. **C**: CK14 antibody stained the full thickness of the epithelium. The signal was more intense along the basal cell layer. **D**: CK4 staining of the SCC epithelium except for the basal layer. **E**: Weak CK3/12 staining of the SCC epithelium. The nucleus was counter-stained blue with DAPI. The original pictures were taken at 200× magnification.

H&E staining revealed thickening of the squamous epithelium of the SCC tissue, as well as local disruption of the basement membrane and epithelial cell invasion of the stroma (Figure 2A,B). The invaded epithelial cells in the stroma showed an enlarged nucleus. Keratin pearls formed by necrotic epithelial cells in the stroma were observed. Massive leukocytes infiltration, stromal fibrosis and irregular collagen fiber deposition in the stroma tissue were also observed.

Immunofluorescent staining analysis showed CK14 expression in all SCC epithelial cells (Figure 2C). CK4 staining was also positive in epithelial cells except for the basal cells (Figure 2D). CK3/12 staining was very weak in the SCC epithelium (Figure 2E).

### Immunofluorescent analysis of S100 proteins in normal corneal-limbal and SCC epithelia

Based on the mRNA abundance, the localization of S100A2, S100A6, S100A8, S100A9, S100A10, and S100A11 proteins was analyzed in normal corneal-limbal and SCC tissue.

**Figure 3:**
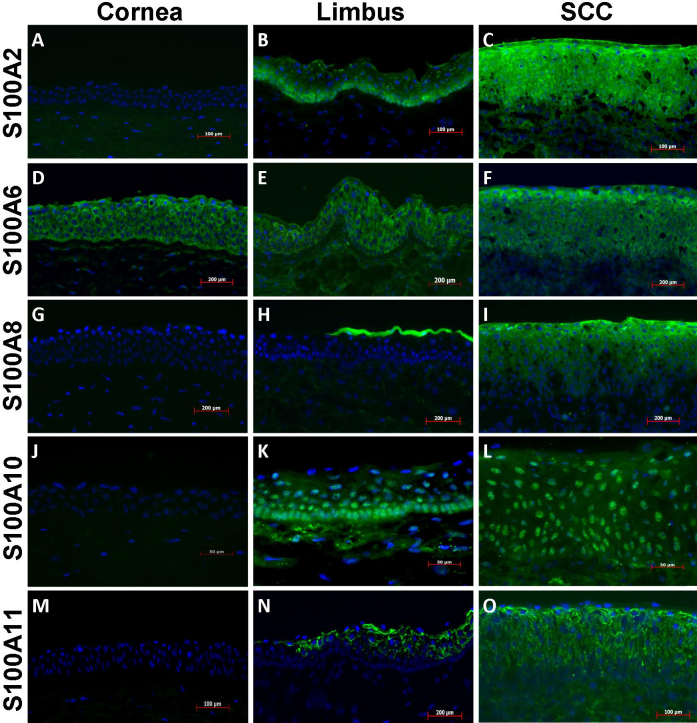
Immunofluorescent staining of S100A2, A6, A8, A10, and A11 proteins (in green) in normal human corneal, limbal and SCC epithelia. The nucleus was counter-stained blue with DAPI. Except for the pictures on S100A10 staining, which were taken at 400×, all others were taken at 200× magnification originally.

S100A2 staining was not observed in normal corneal epithelium (Figure 3A). In contrast, a strong S100A2 staining was consistently observed at the plasma membrane of basal limbal epithelial cells and the staining decreased progressively toward the wing layers (Figure 3B). Sporadic staining was also observed at the superficial layers of the limbal epithelium. The SCC epithelium showed overwhelmingly strong diffusive S100A2 staining in all cells (Figure 3C).

S100A6 staining was observed at the plasma membrane of normal corneal, limbal and SCC epithelial cells (Figure 3D-F). No obvious difference in staining intensity between normal corneal-limbal and SCC epithelial cells was observed.

S100A8 and A9 showed the same staining pattern in the same tissue tested, therefore only the images of S100A8 staining are presented. In normal corneal and limbal tissue, positive staining of both proteins was observed only at the surface of the peripheral corneal and limbal epithelium (Figure 3G,H). However, strong intracellular staining of both proteins was observed in top layers of SCC epithelium. The staining intensity progressively reduced toward the stroma and was negative in the basal epithelial cells (Figure 3I).

S100A10 staining was absent in normal corneal epithelium (Figure 3J). Strong S100A10 staining existed predominately in the nucleus of limbal basal epithelial cells, although some diffusive cytoplasmic staining was also observed (Figure 3K). In addition, S100A10 positive cells progressively decreased toward the upper layers of the limbus. In SCC epithelium, the staining was uniformly concentrated in the nucleus of all cells (Figure 3L).

S100A11 staining was observed at the plasma membrane of superficial layer cells of limbal epithelium adjacent to conjunctiva, but not in the cornea (Figure 3M,N). In SCC epithelia, uniform plasma membrane staining of S100A11 was observed in all cells (Figure 3O).

The localization of S100A proteins in corneal, limbal and SCC epithelia is summarized in Table 2.

**Table 2:** Distribution of S100 proteins in normal corneal, limbal epithelia and SCC tissue.

**Protein**	**Cornea**	**Limbus**	**SCC**
S100A2	Negative	Plasma membrane of basal epithelial cells, decreasing progressively toward upper layers.	Overexpressed in all epithelial cells.
S100A6	Plasma membrane of all cells		
S100A8/9	Negative	Located at the surface of the limbal epithelium.	Top layer cells of the epithelium, decreasing progressively toward the stroma and disappeared at the basal layer.
S100A10	Negative	Nucleus of limbal epithelial cells, decreasing progressively toward upper layers.	Nuclear staining of all epithelial cells.
S100A11	Negative	Plasma membrane staining of upper layer cells adjacent to the conjunctiva.	Uniform staining of all epithelial cells.

### Regulation of *S100A2*, *S100A10*, *S100A8*, and *S100A9* gene expression in cultured limbal epithelial cells

**Figure 4:**
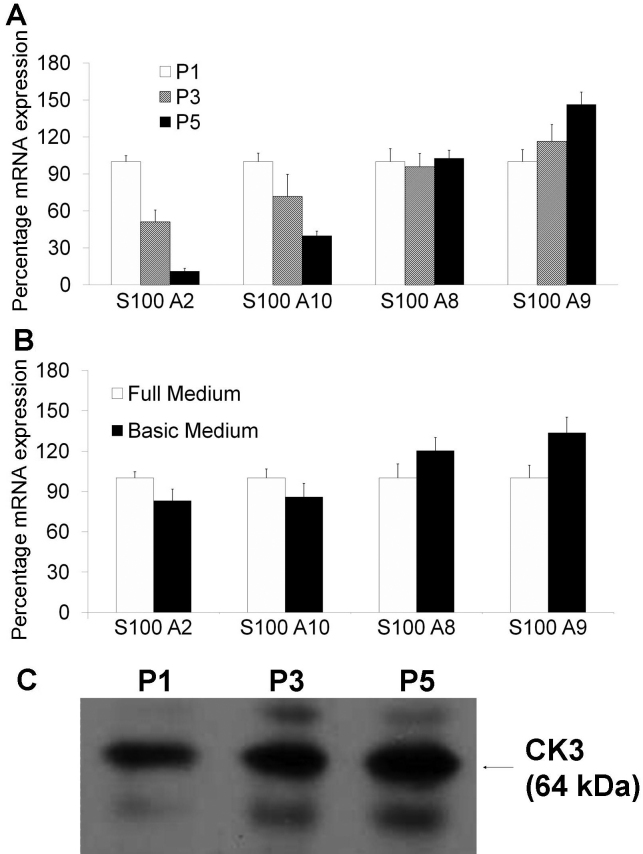
*S100A2*, *S100A10*, *S100A8*, and *S100A9* mRNA expression in cultured limbal epithelial cells. **A**: Fold changes of the above mRNA in P1, P3, and P5 limbal epithelial cells. mRNA level in P1 limbal cells was set as 100%. **B**: Fold changes of the above mRNA in limbal epithelial cells cultured in serum- and growth hormone-depleted medium. mRNA level in cells grown in full medium was set as 100%. **C**: western blot analysis of CK3 protein in P1, P3, and P5 limbal epithelial cells.

Decreased expression of *S100A2* and *S100A10* mRNA was observed in serially passaged limbal epithelial cells (Figure 4A). This is accompanied by a small increase of *S100A9* mRNA, while no significant change of *S100A8* mRNA was observed. Depletion of EGF, insulin, and FBS in the culture medium for 24 h caused a small reduction of *S100A2* and *S100A10* mRNA levels in limbal epithelial cells (Figure 4B). This was accompanied by a small increase of both *S100A8* and *S100A9* mRNA.

Western blot analysis using representative limbal epithelial cell lysates showed increased expression of CK3 proteins in cells with higher passage numbers (Figure 4C).

## Discussion

This study reports the expression of multiple *S100A* mRNA and protein in normal corneal-limbal and ocular surface SCC epithelial cells. These genes were chosen because they are clustered at the epidermal differentiation complex located on chromosome 1q21, a region that contains many genes which encode the structural proteins of the human epidermis [19,20]. We showed the distinctive intracellular localization of S100A2, A6, A8/A9, A10 and A11 proteins in normal corneal-limbal and SCC epithelia. We further provided evidence to show that the expression of *S100A2* and S100A10 is associated with limbal progenitor cells with high proliferation and differentiation capacity. To the best of our knowledge, this is the first study which demonstrated the expression of a large range of *S100A* genes and proteins in normal and carcinoma cells of human ocular surface.

Although all 12 *S100A* gene transcripts examined in the present study were identified in human corneal and limbal epithelial cells, the mRNA for *S100A2*, *S100A6*, *S100A8*, *S100A9*, *S100A10*, and *S100A11* genes were more abundant and the corresponding proteins were detected by immunofluorescent staining. It shall be noted that due to the prolonged transportation time, the corneal epithelium of the cadaver tissue was often damaged with only 1–2 layers of epithelial cells remained when it arrived the laboratory. Therefore corneal epithelial cells collected for qPCR analysis in this study contained a high proportion of basal cells. However, despite the relative high abundance of *S100A2* and *S100A10* mRNA in corneal epithelial cells, immunofluorescent staining analysis showed negative expression of the corresponding proteins in corneal epithelium. On the other hand, we did observe an unusually large variation of ΔCp for many of the *S100A* mRNA examined in both corneal and limbal cells obtained from different donor, indicating that the expression of individual *S100* mRNA may be sensitive to the ocular and tissue culture conditions.

Several S100 proteins, including S100A6, S100A10, and S100A11 bind annexins and are involved in cell membrane organization, ion channel modulation and keratinocyte differentiation [3]. Together with our previous study, we showed that *S100A6* is uniformly expressed in plasma membrane of corneal, limbal, and conjunctival epithelial cells [12]. The staining pattern of S100A11 protein indicated that it is likely associated with CK4-positive conjunctival epithelial cells. This is evidenced by the positive staining of S100A11 in conjunctival epithelial cells as previously reported [12]. In this study, positive S100A11 staining was found only in part of the supralayer limbal epithelial cells adjacent to the conjunctiva. In SCC epithelium, S100A11 positive cells also coincided with CK4 positive cells, while the basal cells of SCC epithelium were negative of both proteins. We speculate that the distinct expression of annexin-binding S100 proteins contributes to the different characteristics of conjunctival and corneal epithelia.

Two S100A proteins were found specifically enriched in the limbal epithelial cells of the normal tissue: S100A2 and S100A10. The cellular localization of these proteins is in agreement with previous findings in other cell types [21-25]. Interestingly, both proteins were found overexpressed in SCC epithelial cells. To test whether *S100A2* and *S100A10* expression was associated with the proliferation and differentiation capacity of the limbal progenitor cells, we examined the expression of these two genes in serially passaged limbal epithelial cells and in cells cultured under serum and growth hormone depleted conditions. Limbal epithelial cells co-cultured with mitomycin-C inactivated 3T3 cells form colonies and are able to maintain a relatively high proliferation potential [17,26]. However, with each passage the cultured cells gradually lose the proliferation capacity and undergo differentiation as indicated by the increasing expression of CK3/12 protein. Significant progressive reduction of *S100A2* and *S100A10* mRNA was observed in limbal cells with increasing passaging. Serum and growth factor depletion also induced a small reduction of both mRNA expressions. Collectively the data suggested that the expression of *S100A2* and *S100A10* is positively associated with the intrinsic proliferation and differentiation capacity of limbal epithelial cells.

S100A8 and A9, also known as calgranulins or myeloid related proteins (MRPs), are mainly expressed in granulocytes and epithelial cells. Secreted S100A8 and S100A9 proteins form heterodimer and are important members of damage-associated molecular pattern proteins (DAMPs) [4,27]. Our results suggested that both proteins were secreted in normal corneal and limbal tissue, similar to what was reported in normal skin epidermis [25]. However, both proteins were overexpressed in SCC epithelial cells and found intra- and extracellularly. This is likely related to the inflammatory conditions associated with SCC as it was also reported in environmentally stressed and inflammatory epidermis as well as in cancer cells [25,28,29]. The changes of *S100A8* and *S100A9* gene expression in limbal epithelial cells under serum and growth factor depleted culture conditions may represent a nutrition-depletion triggered stress response.

Ocular surface SCC is a major cause for ocular morbidity and mortality. Ocular SCC can sometimes develop from CIN, a benign proliferative condition which is more frequently seen than SCC [16]. Finding markers that indicate the progression of CIN and the malignancy of SCC will help clinicians to choose an appropriate therapeutic modality from early on. While abnormal expression of many S100A proteins were found in cancer cells, changes of *S100A2* and *S100A10* expression were reported in various SCC of the oral cavity, esophagus, the larynx, kidney, and the thyroid [30-36]. In this regard, these two proteins may also be candidates for markers associated with the severity of ocular surface SCC.

In summary, this study showed that distinct S100A proteins are involved in the structural and biologic activities of normal corneal and limbal epithelial cells and are associated with ocular surface squamous cell carcinoma. The results presented here warrant further investigation to understand the roles of specific S100A proteins in the regulation of ocular surface epithelial cell proliferation, differentiation and tumor development.
